# The Chlorine and Milk Treatment of Scarlet Fever and the Typhoid Fevers

**Published:** 1862-08

**Authors:** Conway T. Edwards

**Affiliations:** Brompton-row


### THE CHLORINE AND MILK TREATMENT OF SCARLET FEVER AND THE TYPHOID FEVERS.

By CONWAY T. EDWARDS, M.R.C.S.E.

The symptoms, development, and progress of scarlet fever and fever of the typhoid type, have been so continually brought under public notice, and so ably treated in every work on the practice of medicine, that it were superfluous to enter upon a consideration of them in this paper, and would not assist in any way the object I have in view, which is, to point out the beneficial influences which a chlorine and chloroform treatment, with a full diet of milk, exercise over these diseases.

It must not be understood that, in advancing these remedies to so prominent a position in the foreground, they are to be regarded in the light of specifics, or as interfering with the antiphlogistic treatment and regimen which the medical practitioner finds it necessary to adopt during the first stages of febrile disease; but, as possessing the important quality in themselves of at once destroying the putrid effluvia generated by, and thrown of from, the secreting surfaces of the stomach, alimentary canal, kidneys, lungs, and skin, and endowing the system with power to bear the progress of the disease. These effluvia, so poisonous in themselves, on account of the large proportion of sulphuretted hydrogen which they contain, contaminate the air of the sick chamber, oppress nurses and attendants, and are most injurious to the patient. How important, then, must it be to remove them! This chlorine effects; its influence is no question of lengthened time; it commences at once, and continues during the whole course of its exhibition.

Now, as regards "a full diet of milk." From the very commencement of a fever, there is a very great loathing to all kinds of nourishment; the bare suggestion of food creates a shudder, which expresses, more than words can do, how repugnant the idea is to the feelings.

Adopt the once received and re-entertained Brunonian system; give *ab initio* nourishment and tonic medicines, and as the disease advances, with increase of weakness, and ever progressing waste of solids and fluids of the body, perhaps the real nature of the attack may be suddenly developed and resist every remedial means to overcome it. In no disease is the truth of this more apparent than in typhoid pneumonia. Take a false view of the real nature of the attack; see in the fever everything save the current of inflammation which runs beneath it; suddenly the real truth will be developed, and the engorgement of the lungs is but the precursor of the fatal termination which will rapidly ensue. All antiphlogistic treatment would be useless; calomel and opium and antimony have no time to act, and the patient dies.

But the hourly increasing weakness and waste of the system are very formidable symptoms during the course of fever; are these to be ignored, and no attempt be made to meet them? Had we but a life-sustaining diet, simple in itself, and not only agreeable to the patient, but eagerly accepted by him—a diet which in itself should contain meat and drink of the most unstimulating nature—how great a boon it would be to the sufferer!

What writes Dr. Thielman, the Russian physician, in his treatise on Fevers? "He had never seen delirium occur during the progress of *any* fever, under the influence of a full diet of milk, as *after broth!* It is not only well borne, but is easily assimilated, and endues the system with *power to resist* disease." This is exactly what is wanted. How does it agree with our own experience? Yet, without going quite so far, if it only remained on the stomach with ease; relieved the thirst; sustained the nervous energy, thereby giving power to resist the disease,—important results would accrue from it.

It were needless here to enter upon the analysis of milk, or to show forth the reasons why it is the most life-sustaining of nourishments.

It is objected, that milk is indigestible, and therefore not fitted for a weak stomach; that the gastric secretion (analogous to the rennet of the calf) at once separates the solid from the fluid part, the former coming away, especially in children, untouched by the digestive organs. The theory looks well upon paper, but will not bear the test of experience. Curd itself is eaten with pleasure and impunity, not only in the delicious junket, as made to such perfection in Cornwall and Devonshire, but in the more homely form of closely-compressed curd, floating in its whey. Few beverages are more grateful to the palate, more refreshing, and more thirst-allaying than whey: it is the pleasantest and most cooling of summer drinks, and its effects are much more permanent than even water itself.

In giving milk to the fevered system you have at once this most refreshing fluid generated in the stomach, and an unirritating solid formed, which in a greater or lesser degree repairs the waste of the living substance. The gastric juice converts the milk into what is popularly termed "curd and whey."

Now, as to the growth-giving and other nourishing qualities of milk, daily experience proves and shows, in the firm and excellently tinted skin of healthy infants entirely brought up, for seven or eight months, upon milk, its potent and genial influence. The mother, being in good health, supplies a nourishment which no science can equal and no art compound. It is nourishment particularly free from all irritating, clogging, and fever making principle. It is so curiously and wonderfully compounded, that up to a certain point the infant retains it; but beyond that it is rejected, sometimes from over-repletion, on account of its excellent nature, and sometimes from the eagerness with which it is taken.

In treating of milk as a diet especially for invalids, it must be understood that healthy, unadulterated milk is alluded to. This of course is very difficult to be obtained in large towns; almost impossible in the metropolis. In very circumscribed spots, in the latter are assemblages of stalls, capable of holding several hundreds of cows. The impure condition of the heated air is patent to all. It cannot be other than unwholesome. Seldom do the animals scent the fresh air, feel the sunshine, tread the green grass, or take any exercise! Their daily life is spent in eating the most milk-giving, and of course heat-producing, food. In summer, perhaps, long grass, vetches, peahalms, (cut and collected, it may be, for a day or two before use,) are given. In very limited space do they wear away their life in producing milk. The consequences need not be dilated on. The animals are mostly in a fevered state, many are directly diseased, and all produce a milk which (without inquiring too curiously into its composition when retailed to the public) is not healthy. This is not the milk to be selected for a fevered patient, and yet, boiled, it is preferable to a farinaceous or animal soup diet.

We will now turn to the bearing of what has been advanced on scarlet and other fevers.

About the year 1844, scarlet fever raged with some violence in the district where I had practised for many years. At an early period of the attack, typhoid symptoms developed themselves. The dryness of the tongue and fauces, the dark secretion on the gums, putrescent fetor of the breath and from the skin, renal and alvine secretions, attested the dangerous nature of the disease. In Bath, a very extrordinary symptom frequently developed itself, and seemed to be the sure precursor of death. This was a rigidity of the sterno-cleido-mastoidel-muscles, of a nature so great that they felt like rolls of wood beneath the touch; the smaller muscles soon put on the same action, and the sufferer seemed in dying to be strangled. In the country district this formidable complication did not occur; but one family, fearful that an only daughter might take the disease, left for Clifton. Before six weeks had elapsed, this child was seized with the complaint, and died.

In the early days of the epidemic, several fatal cases occurred in my practice, and something akin to a feeling of regret came over me when summoned to any new case. But an accident opened up a mode of treatment, which soon changed the fatal tendency of the attack. Two children, living at the National School, Bathford, took the complaint. They were relatives of one of the nurses at the Bath Hospital, and she came over to nurse them. The symptoms in one child were urgent from the beginning, and both head and chest suffered severely; the throat was studded with sloughing spots, and greatly swollen; the breath and all the secretions gave out the putrefactive fetor. The cases seemed to be of the worst kind. About the fifth day the nurse said: "She has been better, Sir, since she used that last gargle; the great restlessness is gone, the rambling talk has ceased, and all putrid smell from the breath and evacuations has left. I believe," she continued, "it was the gargle which did it, for she swallowed the first dose." This gargle was composed of a solution of the chloride of lime, alum, ustum, compound tincture of iodine, water, and a little tincture of capsicum.

On examining the throat, I found the nurse's statement to be correct as to the breath; and not only had this beneficial change taken place, but the sloughing surfaces were assuming a brighter red, the sloughs were evidently contracting or curling up, and the tongue and gums were becoming moist. There was also a quietude about the look and features, which indicated that the nervous irritability was passing away. The nurse was so positive as to the cause of this favorable change, she was directed to add a drachm of the solution of the chloride of lime to two pints of tepid water, with a little lemon-juice and sugar, and give it as diet-drink to the patient. This, with a gruel and sago diet, and ammoniated effervescent mixture, and an occasional dose of rhubarb and grey-powder and James' powder, were given, until it was safe to administer tonics and good diet. The disease spreading, the same diet-drink was adopted, and the use of chlorine extended by directing the body to be sponged with tepid water containing it, twice a day. After the adoption of this treatment no fatal case occurred.

At the termination of the epidemic, I read a paper on the subject before the Bath and Bristol branch of the British Medical Association, being justified in so doing from the numerous cases which terminated successfully. One or two of the members expressed themselves dubious respecting the effects produced by such means, when the physician who had permitted the nurse to attend on her relatives rose and said that "he could vouch for the accuracy of the writer's statements, for his nurse had narrated to him the results of the treatment." This paper I subsequently published. A week after its publication I was agreeably surprised at receiving a polite note from Mr. Martin Ricketts, of Droitwich, (a total stranger to me,) stating that "he had perused the paper with great interest and pleasure; that he could confirm all that I had written on the subject of the good effects of chlorine in scarlet fever; and that, strange to relate, a similar accident to a child using a chlorine gargle had led to the adoption of chlorine in his practice; lately, however, he had used a preparation made by the Apothecaries' Company at Liverpool, and termed *chloriform*, which appeared to him to possess all the antiseptic powers of chlorine, with a sedative influence peculiarly its own. The dose was from five to thirty minims. He requested my acceptance of a small quantity, regretting at the same time that it was so expensive." In this preparation lay hidden a grand secret—a glorious gem, sparkling, and beckoning to any scientific mind to free it from its prison-house. Its advances were unheeded, until Dr. Simpson first recognized in its component parts something more than the composition of either—a something which possessed all the anæsthetic properties of that fluid without its nauseous ones. What is its position now? It is first on the list of alleviators of human suffering; and the discovery of it should have won for its discoverer a world-wide testimonial. I soon recognized the preparation, made public towards the termination of 1844, to be the same as that sent me by Mr. Ricketts; and, like other medical men, freely experimentalized with it on animals, insects, etc., and then on our suffering fellow-creatures.

On the rebreaking-out of scarlet fever and typhoid epidemic of the usual form, I prescribed chloroform; but as it did not remove the effluvia so effectually as chlorine I administered the latter in combination with it. It was, however, observed that after every dose of chloroform a great calmness and inclination to sleep were produced, unattended with any adverse cerebral symptoms.

The beneficial effects which the use of these remedial means produced in scarlet fever, likewise resulted from their exhibition in typhoid fever; so that I had every reason to be satisfied with the treatment.

But the power of preparations containing chlorine over infectious fevers was known long before the disinfectant principle was discovered to exist in that gas. The celebrated Dr. Parry, of Bath, was an advocate for the use of muriatic acid, both externally and internally, in fevers; but the beneficial influence of that medicine was not referred to the chlorine it contained. That acid is now termed hydrochloric, from its chemical constituents; and in the present day, when the chlorate of potass is freely used in malignant fevers, sore-throats, etc., it is not frequently you find it acknowledged that to the liberated chlorine are the good results attributed. Read any work on fevers, from the school book of the day to the popular *brochure* of the minute, and see what little stress is laid on the value of chlorine as an important curative agent.

Since my residence in London, numerous cases of scarlet fever have come under my care; and, with every disadvantage, in many instances, of impure air, small, ill-ventilated rooms, indifferent diet and nursing, two cases only terminated fatally, and these were seen some days after the disease had first appeared.

It must not be imagined from what has been advanced that these fevers were treated solely with chlorine, chloroform, and milk. The antiphlogistic system was invariably practiced at the commencement of the attack, involving sometimes abstraction of blood, counter-irritation, chloride of mercury, potassio-tartrate of antimony, and purgatives, with gargles of capsicum, alum, iodine, and chloride of lime solution. It is after the first two or three days of this treatment that the internal and external use of chlorine and chloroform is so beneficial, and the milk diet can be depended on—or, if the stomach can bear it, gruel made as thick as good cream, with two-thirds milk; and when we consider what a state of irritation the mucous lining of the primæ viæ must be in, and how certain glandular structures are affected in all typhoid fevers, such a diet cannot but prove most soothing. In conjunction with these, sponging the body twice a day with tepid chlorinated water, change of linen and of bed (where two beds can be placed in the room), will contribute greatly to the general welfare and convalescence of the patient; whilst wine, ale, soups, and jellies can be deferred until the termination of the complaint approaches or urgent symptoms demand them.

I have now brought my observations to a conclusion, with the hope that some other practitioner may be induced to publish his experience of chlorine and chloroform in fevers.—*London Lancet.*

Brompton-row, June, 1862.

